# Problematic cost–utility analysis of interventions for behavior problems in children and adolescents

**DOI:** 10.1002/cad.20360

**Published:** 2020-09-10

**Authors:** Marinus H. van IJzendoorn, Marian J. Bakermans‐Kranenburg

**Affiliations:** ^1^ Erasmus University Rotterdam Rotterdam The Netherlands; ^2^ University of Cambridge Cambridge UK; ^3^ Vrije Universiteit Amsterdam Amsterdam The Netherlands

**Keywords:** cost‐utility analysis, interventions, children, mental health, Quality Adjusted Life‐Years (QALY), EuroQol 5 Dimensions scale (EQ‐5D), critical review

## Abstract

Cost–utility analyses are slowly becoming part of randomized control trials evaluating physical and mental health treatments and (preventive) interventions in child and adolescent development. The British National Institute of Health and Care Excellence, for example, insists on the use of gains in Quality Adjusted Life Years (QALYs) to compute the “value for money” of interventions. But what counts as a gain in quality of life? For one of the most widely used instruments, the EuroQol 5 Dimensions scale (EQ‐5D), QALYs are estimated by healthy individuals who provide utility scores for specific health states, assuming that the best life is a life without self‐experienced problems in five domains: mobility, self‐care, usual activities, pain/discomfort, and anxiety/depression. The worst imaginable outcome is defined as “a lot of problems” in each of these five domains. The impact of the individual's problems on the social network is not weighted, and important social–developmental domains (externalizing problems, social competence) are missing. Current cost–utility computations based on EQ‐5D favor physical health over mental health, and they rely on adult weights for child and adolescent quality of life. Thus, a level playing field is absent, and developmental expertise is sorely missing.

For policy advisers and politicians, health economics is increasingly important because of cost–utility analyses of randomized control trials (RCTs) on mental health problems. A crucial question is whether and when they are willing to pay the price of investment in child and adolescent interventions. In particular, we focus here on the costs of interventions aiming at behavior problems and lack of social competence in return for benefits enjoyed by children, their parents, and society. In its report on “Judging whether public health interventions offer value for money” the National Institute of Health and Care Excellence (NICE, 2018) in the United Kingdom, for example, recommends the use of Quality Adjusted Life Years or QALYs to compute the value for money of interventions. In health economics, other approaches have been developed to estimate costs and benefits of interventions, and the willingness to pay for a treatment, for example, the Disability Adjusted Life Year or DALY approach (e.g., McBain et al., 2016) but they are beyond the scope of the current paper (see Drummond, Sculpher, Claxton, Stoddart, & Torrance, 2015, for other methods). In this paper, we discuss the scientific, normative, and developmental assumptions of one of the most widely used health economics models as currently applied to child and adolescent (preventive) intervention programs. The EuroQol 5 Dimensions scale (EQ‐5D) used to retrieve the utility scores to compute QALYs is taken as an example to illustrate the challenges of a predominant health economics approach. Health economics is an emerging field of inquiry that cannot be left to economists alone. Developmental science expertise should be brought to bear on the premises, measurements, and analyses in cost–utility computations, lest children, adolescents, and their families suffering from mental health issues pay the price.

## 1 COST–UTILITY ANALYSIS

Cost–utility analysis is often defined as the evaluation of “the impact of the intervention in terms of improvements in preference‐weighted health‐related quality of life, such as the Quality Adjusted Life Year (QALY)” (Beecham, 2014; p. 715). A basic idea is that it is possible to create one common yardstick or generic currency across all health‐related interventions. The QALY would be such a currency: the gold standard against which any health‐related intervention in any clinical or at‐risk group could be measured, allowing decision‐makers to select the prevention or intervention program with the best cost/quality ratio. Based on QALY, it would even be possible to weight the best value for money of medical treatments against that of mental health interventions, a truly interdisciplinary ambition.

The idea behind QALY was introduced in a paper by Klarman, Francis, and Rosenthal (1968) on the advantages of transplantation versus dialysis in patients with renal failure (see also MacKillop & Sheard, 2018). In this paper Klarman not only took into account the number of years of life added by each of the treatments, but also the quality of these extra years of life after transplantation, which was estimated to be 25% better than with dialysis. In QALY, the combination of quantity (mortality) and quality (morbidity) of life years is the core of the computation. It is evident that patients experience life after transplantation as “better,” compared to life with regular dialysis. But how did Klarman et al. (1968) arrive at the 25% quality bonus? In this case, they just assumed without any further theoretical or empirical evidence that the extra life quality could be quantified as one quarter of each life year gained.

### 1.1 Creating a gold standard for quality of life

A more sophisticated way to estimate quality of life has been developed by assessing patients’ or non‐patients’ preferences for a shorter life without health issues, versus a longer life with health problems lowering the quality of the extra years. The preferences are established on the basis of a generic measure of quality of life that covers various domains of functioning such as physical, mental, and social functioning. For example, the widely used instrument EQ‐5D (see EuroQol, 2020) comprises five questions, covering five dimensions, that is, physical mobility, looking after yourself, doing usual activities, having pain or discomfort, and feeling anxious or depressed. In the EQ‐5D‐Y (Youth version; see EuroQol, 2020), the questions have been slightly adapted, using, for example, the terms worried, sad, or unhappy instead of anxious or depressed. Each dimension has three levels corresponding to (1) “no problems,” (2) “some problems,” and (3) “a lot of problems.” The various health states are indicated by one of the three scores on the five dimensions (e.g., if the respondent indicates to experience a lot of problems in the first dimension, mobility, and some depressive problems but no problems in the other dimensions, the health state is represented as 31112. In an updated measure, the EQ‐5D‐5L (see EuroQol, 2020), the same five dimensions are used with more differentiated response alternatives (no, slight, moderate, severe, and extreme). Validation of this revized measure is underway but, currently, NICE still recommends the three‐level utility estimates as a basis for computing QALYs.

One way to establish preferences or utilities for each of the various health states is to survey a large representative sample asking the respondents to choose between extra life years with the impairments in each of these dimensions (*t*) and a shorter life without any problems on these dimensions (*x*). This time trade‐off (TTO) approach results in a preference or utility score for the impaired status of *x*/*t*. In other words, the respondents are asked to indicate the number of years in full health that they consider equivalent to 10 years in a specific impaired state (e.g., with a lot of problems in the mobility dimension and some problems with depression, state 31112). When a respondent considers 6 years in full health equivalent to 10 years with a lot of mobility problems and some depressive problems, the respondent's weight for this state would be 6/10. Based on a sample of respondents answering the same question their average opinion is the weight for that state of (ill) health which subsequently is used to compute the QALY. The approach fits the traditional economic model of the “homo economicus” who is a rational respondent with preferences (or utilities) with the goal to maximize these individual, self‐interested preferences (Melé & Cantón, 2014).

### 1.2 From full health to death

In fact, the weights are rankings on a continuum from zero (death; state 33333) to one (full health; state 11111). In a UK representative sample of 2,997 non‐clinical adult participants, Dolan, Gudex, Kind, and Williams (1995) used the TTO approach to valuate the various states. The participants showed their preferences (utilities) for a large number of states, which resulted in coefficients for levels 2 (some problems) and 3 (a lot of problems), respectively, for each of the domains, see Table 1. The algorithm is completed with a constant: .081, and a “malus” of −.269 when one or more score of 3 (a lot of problems) on any dimension is given. Dolan et al. (1995) calculated the constant as the intercept of the regression equation that is at the basis of the computation of utilities for the various health states, and it indicates any deviation from perfect health. The malus is also a constant that Dolan et al. (1995) included in the regression equation to avoid the residuals to be associated with the predicted values. The relevant weights for a specific state are subtracted from the state of full health in all domains. Thus, state 11111 amounts to a weight of .919 (the constant .081 subtracted from 1), and the weight for the worst state 33333 (“death”) amounts to −.594. About one third of all health states receive weights lower than 0 (Devlin, Shah, Feng, Mulhern, & Van Hout, 2018).

**TABLE 1:** Time trade‐off (TTO) weights for the various health states represented by EuroQol 5 Dimensions Scale (EQ‐5D) to compute quality adjusted life years (QALYs)

	Mobility	Self‐care	Activity	Pain	Depression
Some problems	.069	.104	.036	.123	.071
A lot of problems	.314	.214	.094	.386	.236

Constant .081; malus for “a lot of problems” −.269.

Weights derived from Dolan et al. (1995).

This approach leads to “social tariffs” for each condition or health state, and these tariffs are being used in health economics studies across the world and for every age cohort since 25 years (e.g., Goodyer et al., 2017). The state indicated by 31112, for example, gets the QALY weight of 1 − .081 − .314 − 0.000 − .000 − .000 − .071 − .269 = .265. Once the weights for each response category and the social tariffs for each health state are established in a large survey like Dolan et al. (1995)’s study, participants in a RCT can be asked to answer the five questions, and the QALYs for each of the subjects can be computed. If participants after treatment or intervention find themselves in a health state that is higher (closer to full health) than participants in the control group who received care as usual, the intervention is considered to be better than care as usual. The expenses for the intervention compared to care as usual can be summarized and cost per QALY gain is computed. Interventions with lower cost per QALY gain will in general be preferred above those with higher costs—assuming sufficiently replicated evidence for robust positive effects of the preferred intervention.

The QALYs are thus generic in the sense that they can be used for the evaluation of any treatment or intervention in the developmental, social, psychological, medical, psychiatric, clinical, or preventive domain because the five dimensions pretend to cover the whole gamut of components of the “good life” (Drummond et al., 2015). In principle, all health‐related interventions and treatments could be listed in one ranking of more to less value for money (Dixon & Welch, 1991). It should be noted that because no valuation for the child and adolescent EQ‐5D‐Y has yet been conducted (https://euroqol.org/eq-5d-instruments/eq-5d-y-about/valuation/) the weights assigned to each state of child or adolescent health are currently the same as the adult weights, although considerable doubts about this generalization can, and have been raised (Kind, Klose, Gusi, Olivares, & Greiner, 2015).

The Child Health Utility‐9 Dimensions scale (CHU‐9D) with nine questions covers a somewhat broader range of functioning (worry, sadness, pain, tiredness, annoyance, school, sleep, daily routine, and activities; Furber & Segal, 2015) but similar to the EQ‐5D‐Y it also lacks externalizing and social competence dimensions. The CHU‐9D seems more tailored to adolescent development than the EQ‐5D‐Y, although adult tariffs for CHU‐9D have often been used (Stevens, 2012). An additional issue is that the reliability and construct and convergent validity of the CHU‐9D scores seem rather modest (Stevens, 2012). Here we focus on the EQ‐5D‐Y, but developmental analysis of the CHU‐9D approach is critically needed.

### 1.3 QALY as the gold standard

QALY has become the gold standard in health economics and policy, in the UK as well as in other Western countries. The report on “Judging whether public health interventions offer value for money” (NICE, 2018) considers an intervention good value for money if the cost of an intervention that manages to create one QALY gain is less than £30,000. It is argued that in any society the budget for health‐related interventions will be limited, scarce resources have to be distributed, and budget constraints will be set by politicians. Within these budget limits policy makers might feel obliged to choose an evidence‐based treatment for depressive adults of £10,000 per QALY gain, instead of a preventive intervention reducing emerging conduct problems in children for £12,000 per QALY gain. A childhood intervention for post‐traumatic stress symptoms that would cost £50,000 per QALY gain would not be fundable except when policy makers want to take into account other than only budgetary considerations such as a strong patient lobby or firm public opinion about the need for such an intervention.

It should be noted that weights for the various states might differ between countries, even when they are neighboring countries with rather similar cultures. For example, in one of the few countries with their own weights, the Netherlands, respondents assign more weight to the depression and anxiety dimension compared to the UK participants, and less weight to the other dimensions (Lamers, McDonnell, Stalmeier, Krabbe, & Busschbach, 2006). No utility scores for computation of QALYs are available from valuation studies in most lower and middle income countries (LMICS). In these countries the DALY approach is more often used (e.g., McBain et al., 2016). The World Health Organization recommends the DALY method for LMICS interventions, defining a DALY as one lost year of “healthy” life (https://www.who.int/healthinfo/global_burden_disease/metrics_daly/en/). Because DALYs have major impact on policy decisions in LMICS, cross‐cultural anthropological and sociological knowledge about child and adolescent development acquired in the developmental sciences is critically needed. Here we limit ourselves to the QALY approach.

## 2 CHILD AND ADOLESCENT MENTAL HEALTH ECONOMICS IN PRACTICE

**FIGURE 1:**
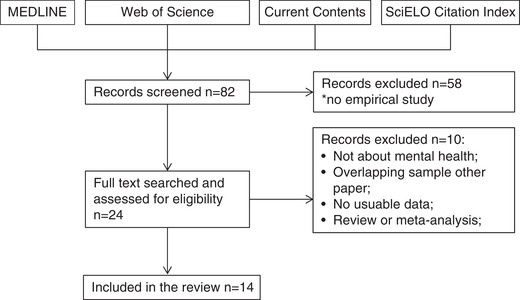
Flow chart of the literature search of randomized control trials focusing on infant or child mental health issues using health economics, in particular the QALY approach

In order review to how the cost–utility analysis has been applied in intervention studies on child and adolescent mental health we searched in Web of Science, Current Contents, Medline, and SCIELO for empirical papers presenting RCTs focusing on infant or child mental health issues using health economics, in particular the QALY approach. Search terms were (“cost‐effectiveness” or “cost‐utility” or QALY or “health economics”) AND (infan* or child*) AND (“mental health” or “behavior problem*”) AND (“randomised trial*”). The search terms for mental health and behavior problems are quite broad and treatments targeting a specific disorder might not have been included. We focused on the most common issues and interventions. Figure 1 presents the search (*k* = 14 studies; March 3, 2020).

The use of QALYs in economic analyses of mental health interventions is relatively recent, and only a small proportion of randomized control trials present a cost–utility analysis. But the number of pre‐registered protocols for RCTs which include a health economics component is growing. In the past 7 years, 14 RCTs on child and adolescent mental health analyzed cost–utility or value for money. The studies are summarized in Table 2. From Table 2 it may be derived, for example, that the Canaway et al. (2019) school‐based intervention program “Waves” targeting obesity required an investment of 155 pounds per child (ages ranging from 6 to 7 years.) and that this intervention resulted in .006 QALY gain. The incremental cost‐effectiveness ratio (ICER) thus amounted to 26,815 pounds. The QALY gain calculation was based on the CHU‐9D and the cost‐effectiveness of Waves can now be compared with any other intervention targeting obesity or any other (mental) health issue. Most randomized trials were group based, and focused on adolescents with internalizing issues such as depression, anxiety, insomnia, obesity, and PTSD (e.g., Canaway et al., 2019; Robertson et al., 2017). In most studies, the intervention or treatment was compared to care as usual (e.g., Ougrin et al., 2018; Simkiss et al., 2013; Turner, Carter, Sach, Guo, & Callaghan, 2017), but some studies compared two or more different treatments (e.g., Chatterton, Rapee, & Catchpool, 2019; Creswell et al., 2017; De Bruin, Van Steensel, & Meijer, 2016; Sayal et al., 2016).

**TABLE 2:** Randomized control trials focusing on child or adolescent mental health issues using health economics, in particular the QALY approach

Author	*N*	Age (years)	Focus	Intervention program	Type of intervention	Added costs	QALY gain	Cost/QALY (ICER)	QALY basis
Canaway et al. (2019)	1397	6–7	Obesity	Waves	School based	£155	.006	£26,815	CHU‐9D
Creswell et al. (2017)	136	5–12	Anxiety	CBT vs. solution‐focused therapy	Home based + phone	£448	.006	£74,667	CHU‐9D; EQ‐5D‐Y
Goodyer et al. (2017)	465	11–17	Depression	CBT vs. STPP vs. BPI	Clinic	£904	‐.009	n.a.	EQ‐5D
Ougrin et al. (2018)	106	11–17	Inpatients	SDS	Clinic	£24,000	.000	£183,750	EQ‐5D
Robertson et al. (2017)	116	6–11	Obesity	Family for Health	Family based	£450	.04	£552,175	EQ‐5D
Sayal et al. (2016)	199	4–8	ADHD	Patchwork	Group family + teacher	£193	−.028	‐£33,751	EQ‐5D‐Y CHU9D
Simkiss et al. (2013)	286	2–4	Universal	Family Links Nurturing Programme	Group family	£648	.019	£34,913/5 years	SF‐6D
Turner et al. (2017)	86	14–17	Depression	Physical exercise	Group	£589	.0019	£152,822	EQ‐5D‐5L
Anderson et al. (2014)	3357	12–16	Depression	CBT RAP	Group school	£142	−.05	n.a.	EQ‐5D
McBain et al. (2016)	436	15–24	War affected	Youth Readiness Intervention	Group	$104	.01	$7260	DALY → QALY
De Bruin et al. (2016)	62	16	Insomnia	CBT group vs. internet	Group	n.a.	.00	n.a.	EQ‐5D
Barnes et al. (2017)	166	16–24	Maltreatment	Group Family Nurse Partnership	Group	£2072	−.01	−£247,485	EQ‐5D‐5 L
Shearer et al. (2018)	29	8–17	PTSD	Cognitive Therapy	Individual	£1463	.0087	£2,205	SDQ → CHU‐9D
Chatterton et al. (2019)	281	7–17	Anxiety	Stepped care vs. Cool Kids	Individual	$328	−.011	n.a.	AQOL‐8D + CHU‐9D

*Note*. “Universal” means that the intervention is not focusing on a specific group but on the general population.

Abbreviations: CBT, Cognitive Behavioral Therapy; STPP, short‐term psychoanalytic psychotherapy; BPI, brief psychosocial intervention; SDS, supported discharge service; SF‐6D, Short‐Form Health Survey with six dimensions; RAP, Resourceful Adolescent Programme; AQOL‐8D, the Assessment of Quality of Life—eight‐dimension scale.

QALY assessments were conducted with the EQ‐5D and the CHU‐9D. One study used the mapping of the Strengths and Difficulties Questionnaire (SDQ) on CHU‐9D to get utility weights (see Section 3.7 (Shearer et al., 2018). The primary outcome effects of most studies were non‐significant, as were the effects on QALYs, with the exception of one trial on the effectiveness of cognitive therapy for PTSD (Shearer et al., 2018). In four trials, negative QALY effects were found (Anderson et al., 2014; Barnes et al., 2017; Goodyer et al., 2017; Sayal et al., 2016). It is difficult to see the relevance of a cost–utility analysis in the absence of a QALY gain of the intervention, except maybe when a small negative effect is outweighed by much lower expenses compared to care as usual. From an ethical perspective, an intervention that lowers quality of life might be considered iatrogenic compared to care as usual, and thus inadmissible even with cost savings.

The incremental cost‐effectiveness ratio (ICER) for most of these interventions was above conventional criteria for willingness to pay (£20,000–30,000). This is a bleak picture of the cost–utility in a set of 14 recent randomized trials aimed at improving mental health of children and adolescents. This is even more remarkable when the relatively low added costs for the interventions is taken into account, with most of the interventions costing less than £1,000. Furthermore, most interventions were implemented on the group level, and interventions on the individual level will almost always be costlier. This implies that individual treatments will tend to present even more negative ICERs, unless they are much more effective. In this set of trials, QALY gains seem small. Largest gains might be produced by interventions focusing on one severe handicap: When an intervention manages to lower the severity of this handicap from 3 (a lot of problems) to 2 (some problems) with everything else remaining equal, this would computationally mean that the malus of −.269 would turn into a bonus of .269. The downside is that (preventive) interventions aiming at reducing “some” problems in most or all domains would automatically show less improvement in QALYs, because the malus is not included in a pre‐test valuation. In other words, the gap between 3 (a lot of problems) and 2 (some problems) is much larger than the gap between 2 and 1. An update of the EQ‐5D measure to the EQ‐5D‐5L with five instead of three steps in response categories might partly address this problem.

## 3 THE IRRATIONALITY OF QALYs

We argue that for several reasons, the health economics analyses of interventions in the area of child and adolescent development are based on untenable assumptions and wrong standards in terms of age and domains of health and happiness. One of these untenable assumptions is the idea that individuals would only strive for maximizing their own health and happiness.

### 3.1 Homo economicus?

In the EQ‐5D approach QALYs are defined and weights are computed for each of the five domains with the assumption that healthy, rational individuals aim at maximizing their individual utilities. They decide whether they prefer a specific number of life years with full health in each of the five domains to life years with “some” or “a lot” of problems in one or more of these domains. It is argued that the sample should consist of healthy individuals because respondents with a specific clinical disorder, such as chronic severe pain, would “overrate” this component of a healthy life.

Empirically, QALYs as determined by EQ‐5D are dominated by indicators of physical health as opposed to mental health: physical mobility, self‐care, and having pain or discomfort are all mainly related to physical health, whereas only depression is specifically related to mental health. Being able to do usual activities might be assigned to both the physical and mental health domains. Moreover, only mental health problems of the internalizing kind—depression and anxiety—have been included, and externalizing issues such as aggression and conduct problems have been left out. The ideal individual, that is, the optimal individual state is defined as a healthy and happy person. However, the implication is that it is deemed unimportant whether this individual can regulate negative emotions and aggressive behaviors, or whether the person shows prosocial behavior.

### 3.2 Rational decision‐maker?

The TTO approach might appear to be a rational decision procedure between the various states defined as less than full health, but the resulting weights for the various states are far from rational choices. A healthy individual of 25 years old without having experienced any of the problems in any of the five domains cannot rationally decide between one and another set of issues. This individual does not really know what physical pain is, or what it means to have a major depression with suicidal thoughts. According to the QALY weights, “a lot” of problems with depressive or anxious feelings (.236) are weighted less than “a lot” of problems in the domain of pain or discomfort (.386). Without having experienced both, these states cannot be balanced against one another, and the consequences of a decision cannot be calculated in a rational way. Restrictions in life experiences or lack of empathy with the future self who might suffer from pain or depression limit the validity of such decisions.

Moreover, different ages might lead to different weights, as septuagenarians might weight lack of mobility and self‐care much higher than millennials (Szende, Janssen & Cabases, 2014). At the same time, they might be less inclined to exchange healthy years for years with incomplete health because their life expectancy is much shorter than that of a millennial. The valuation of about one third of the ill‐health states as worse than death might be understandable from the perspective of the social network around the individual but the concept of death is extremely complicated to grasp for older individuals, and even more difficult for individuals at younger ages with less loss through death experiences (Chisholm, Healey, & Knapp, 1997). For these reasons, among others, it is even more irrational to use adult weights for the computation of QALYs of children or adolescents (see also Rowen et al., 2020).

### 3.3 Parent as proxy?

For children unable to answer the five questions about their physical and mental health because of age or mental abilities, it has been suggested that a proxy such as a parent might complete the questionnaire about problems experienced by the child from the perspective of the child. This is a biased, non‐rational procedure for at least two reasons. First, attachment research has shown that quite a few parents are insensitive to their children's feelings of stress and distress and try instead to avoid or dismiss such negative emotions because they trigger their own childhood distress experiences (Bowlby, 1969). Poverty has been shown to lower sensitivity of parents for their children (Bakermans‐Kranenburg, Van IJzendoorn, & Kroonenberg, 2004) as parents’ main focus is forced to be on coping with adversities and survival. Second, parents and children do not necessarily have the same interests in health and happiness for the child. Parents may have their own goals in terms of the child's health and happiness, for example, a desire for an uninterrupted sleep despite the infant's need for feeding during the night. From an evolutionary inclusive fitness model, Trivers (1974) showed that parents are inclined to distribute scarce resources between their offspring, whereas each child tries to maximize parental care and its chances of survival, if needed at the expense of his or her siblings. Based on their systematic review of convergence between self‐reported and proxy‐reported assessments of utilities, Khadka et al. (2019) concluded that we should mind the rather wide inter‐rater gap (see also Kwon et al., 2018).

### 3.4 Development is a missing link?

From a developmental perspective the use of adult weights in the calculation of QALYs is, of course, a major problem. Human development is not a static but dynamic and transactional phenomenon (Barbot et al., 2020, this issue) which changes in its wake drastically the meaning and substance of a good life of “health and happiness.” In infancy large part of the social world are the parents or other main caregivers who protect the child and take care of the basic physical needs but also stress regulation and security, and the parents’ well‐being converges with that of the infant. In adolescence the role of peer relationships becomes more important with related challenges of social rejection and aggression, and “health and happiness” will have to be operationalized in a different way. Furthermore, a major issue of the way in which QALYs are calculated is that they consider utility as a static numerator although it is often unknown what the long‐term effects of interventions are, in particular for developing youth with changing adaptational challenges. High QALY gain of a perinatal intervention with parents might dissipate in early adolescence where developmental demands on the parents interacting with their children striving for independence will be rather different. In the TTO approach the time window is 10 years but a decade in a child's life is of a different level than the same 10 years in the life of a 40‐year‐old adult. Accounting for such developmental differences might be complex but is required if the QALY approach is being used to evaluate cost‐effectiveness of preventive measures, interventions, and treatments in childhood and adolescence.

### 3.5 Level playing fields?

With the EQ‐5D for QALY assessments, mental health intervention and prevention programs cannot compete for scarce resources on a level playing field with medical health prevention or intervention efforts. The first reason is that the mental domains of (dis‐)functioning are underrepresented in the catalogue of five domains for the computation of QALYs. The second reason is that the physical domains have been assigned larger impacts. For example, a lot of problems with pain or discomfort which interfere with daily life compared to a lot of problems with depressive or anxious feelings interfering with daily life lead to different QALY weights. State 11331 (disabling pain or discomfort) is weighted .17, whereas state 11313 (disabling depression or anxiety) is weighted .32. An effective pain intervention leading to state 11221 (weight .76) would result in a .59 QALY gain (.76 − .17 = .59), whereas an effective depression intervention leading to state 11212 (weight .812) results in a QALY gain of .492 (.812 − .32 = .492). A medical pain intervention can thus be 10% more expensive than a psychological intervention for depression to be equally fundable for QALY‐oriented policy makers.

### 3.6 Homo socialis?

A serious problem is the absence of the externalizing domain (Gintis & Helbing, 2015). Evidence‐based parent–child interventions such as VIPP‐SD (Juffer, Bakermans‐Kranenburg, & Van IJzendoorn, 2017), ABC (Bernard et al., 2012), and Incredible Years (Gardner et al., 2019; O'Neil, McGilloway, Donnelly, Bywater, & Kelly, 2013) aim at reducing or preventing externalizing behavior problems like aggression, conduct, and oppositional problems. However, when the effects of these interventions are assessed with the EQ‐5D in which the main intervention goal of decreasing externalizing problems is missing, no QALY gain can be made. Nevertheless, it is clear that such problems have a significant negative impact on the individual as well as on his or her social network and society at large. From the current health economics perspective on quality of life, only the individual's self‐centered health and happiness are taken into account, not the potential positive or grave consequences for others. For example, the cost–utility analysis of a parenting intervention program might be conducted with QALY gains for the child but the effects of these gains on parents who experience less stress and exhaustion are discounted. The same goes for positive effects on siblings within the family (Mortimer & Segal, 2006). Similar ripple effects into the future school life of the children who become more self‐regulated in their behavior in the classroom are neglected as well (Belsky, 2009).

### 3.7 Mapping algorithm?

Without child‐generated data on a QALY measure the only method currently available to compute QALYs for children is mapping some existing assessment of problem behavior on the EQ‐5D‐Y or a similar approach (e.g., the CHU‐9D) (Mukuria et al., 2019). In one study, 200 caregivers of 5–17‐year‐old Australian children receiving community mental health services completed the widely used (SDQ (Goodman, 2007) and the CHU‐9D in a telephone interview (Furber, Segal, Leach, & Cocks, 2014). The CHU‐9D utility value was estimated as a linear multiple regression function of the five SDQ subscales for emotion, conduct, peer relations, prosociality, and hyperactivity. The SDQ explained about 28% of the variance in CHU‐9D scores. The regression function for this SDQ‐based utility = .880 + (−.019 × emotion) + (−.009 × conduct) + (−.001 × hyper) + (−.008 × peer) + (.005 × prosocial).

Positive features of this proximal utility function are the inclusion of externalizing issues and the prosociality subscale that uniquely contributed a positive, albeit negligible utility weight. However, it does not make good sense to use this substitute for the original QALY measure because only a small percentage of the variance is being explained, and the small sample cannot be taken as representative of the general (clinical or non‐clinical) population in the wide age range of 5–17 years (Shearer et al., 2018). Extrapolating the proximal utility function to the ages 0–5 years is even more problematic.

## 4 CONCLUSION

Economic cost–utility analyses are becoming part, and parcel of RCTs aiming at a better life of children, adolescents, and their families. The crucial question is what “a better life” means from a traditional health economics perspective. QALYs as measured with the EQ‐5D define the “best life” as a life without self‐experienced problems in the five domains of mobility, self‐care, usual activities, pain/discomfort, and anxiety/depression, and the “worst life” as a lot of problems in each of these five domains. The impact of problems on the patient's social network (e.g., family, peer group, classroom, neighborhood) is not taken into account, and important social domains, such as the domain of externalizing behavior problems and prosociality, are missing. Current cost–utility approaches favor physical health over mental health, and they rely on adult weights for child and adolescent quality of life. Thus, a level playing field is absent. The equation “value for money” requires not only insight into the pecuniary concept of “money” but also a developmental perspective on the “value” component.

Here we discussed the use of the EQ‐5D, one of the most widely used instruments in computing value for money in terms of QALYs. Interdisciplinary discussions and studies on psychometric, ethical, and developmental assumptions of this specific measure and alternative approaches in health economics are needed across the developmental, health, social, anthropological, and economic sciences. The ultimate aim is to promote a better life for children, adolescents, and their families even when resources are scarce.

## AUTHOR CONTRIBUTIONS

Marinus H. van IJzendoorn conducted the literature search, data collection, and data analysis. Marian J. Bakermans‐Kranenburg and Marinus H. van IJzendoorn contributed equally to figure, study design, data interpretation, and writing. Both authors approved the final manuscript.
